# Point‐of‐care nucleic acid testing – a step forward in controlling the HIV epidemic: A review

**DOI:** 10.1111/hiv.13757

**Published:** 2025-01-25

**Authors:** Suleyman Sarp Pinar, Mark Manak, Shanmugam Saravanan, Nesrina Imami, Catherine Kibirige

**Affiliations:** ^1^ Imperial College School of Medicine, Imperial College London London UK; ^2^ Turesol Consulting King of Prussia Pennsylvania USA; ^3^ Saveetha Medical College and Hospital, Saveetha Institute of Medical and Technical Sciences (SIMATS) Chennai India; ^4^ Centre for Immunology and Vaccinology, Imperial College London London UK

**Keywords:** diagnostics, HIV, nucleic acid, PCR, point‐of‐care, treatment monitoring

## Abstract

**Introduction:**

The HIV/AIDS epidemic, with 85.6 million infections and 40.4 million AIDS‐related deaths globally, remains a critical public health challenge. Current diagnostic methods, primarily fourth‐generation immunoassays, have limitations due to their long window periods, and most viral load assays require centralized testing protocols that result in delays, especially in remote regions.

**Nucleic Acid Testing:**

Point‐of‐care (POC) nucleic acid amplification testing (NAAT) presents a transformative approach by reducing the window period for detection to one week and significantly shortening turnaround times for viral load monitoring.

**Discussion:**

This review highlights the clinical utility of POC NAAT for acute HIV infection diagnosis, its role in timely combination antiretroviral therapy adjustments, and its potential to reduce the basic reproduction number (*R*_0_), a critical threshold for suppressing the epidemic.

**Conclusion:**

By improving early detection and facilitating faster clinical decisions, POC NAAT enhances the effectiveness of HIV prevention and treatment programmes, particularly in high‐risk and remote communities, and supports the global effort to achieve the ambitious UNAIDS 95‐95‐95 targets.

## INTRODUCTION

Infection with HIV, a lentivirus descendant of the simian immunodeficiency virus (SIV), is a progressive, incurable disease which attacks the immune system and ultimately leads to AIDS [1]. Untreated HIV‐positive patients progress to AIDS within the first decade of diagnosis, and most (52%) die of an AIDS‐related illness (e.g. opportunistic infections or malignancies, etc.) within 2 years of the onset of AIDS [2]. Since the first cases were reported in 1981, the HIV/AIDS epidemic has spread rapidly throughout the world, with approximately 85.6 million people currently infected with HIV and an estimated 40.4 million succumbing to AIDS‐related illnesses [3].

Third‐generation tests, which are typically enzyme‐linked immunoassays (EIAs) that detect HIV antibodies, have a window period of approximately 3 months. The window period is the time between potential HIV exposure and the point at which a test can reliably detect the virus. Currently, the widely used diagnostic test algorithm for screening HIV infection employs fourth‐generation immunoassays, which simultaneously test for p24 antigen, a protein produced early during HIV infection, and immunoglobulin M (IgM) and IgG antibodies to HIV‐1 and HIV‐2 [4], followed by the Bio‐Rad Geenius assay for HIV‐1 and HIV‐2 differentiation [5]. This has enhanced the diagnostic accuracy of the tests and reduced the window to ~2 weeks [4, 6]. This early detection allows for the earlier initiation of treatment, which can significantly reduce the risk of HIV transmission and improve long‐term health outcomes.

HIV became a chronically manageable condition with the administration of a combination of small molecules that suppress viral replication, referred to as combination antiretroviral therapy (cART) [7]. WHO‐recommended first‐line cART in sub‐Saharan Africa, for example, consists of two nucleoside reverse transcriptase inhibitors (NRTIs), such as tenofovir disoproxil fumarate (TDF) and lamivudine (3TC) or emtricitabine (FTC) combined with one non‐nucleoside reverse transcriptase inhibitor (NNRTI), such as efavirenz (EFV) or nevirapine (NVP), and one integrase inhibitor, such as dolutegravir (DTG) [8]. However, due to the high incidence of cART resistance, patients' viral load (VL) must be periodically monitored, as a VL consistently above 200 HIV‐RNA copies/mL may indicate cART resistance [9]. VL testing, a form of nucleic acid amplification testing (NAAT), is currently undertaken within one or a few central laboratories in most sub‐Saharan countries, which collectively harbour over 65% of the people living with HIV worldwide, resulting in a long turnaround for HIV‐treatment monitoring results in remote communities in this region [10, 11, 12, 13].

Nucleic acid amplification testing is a molecular technique used to detect very low levels of viral RNA or DNA in samples by amplifying their genetic material [14]. Point‐of‐care (POC) NAAT is a powerful tool for acute HIV infection diagnosis, as it reduces the window period for HIV diagnosis to 1 week [15] and provides test results at the point of patient care. Greater access to POC NAAT in remote settings, particularly in sub‐Saharan Africa, will greatly impact HIV treatment and care because these areas have the longest test result wait times when the current predominantly centralized systems are utilized [10]. Therefore, this review article will focus on the clinical utility of POC NAAT in remote communities [14].

## ACUTE HIV INFECTION DIAGNOSIS WITH NAAT

HIV infection begins with acute infection marked by high viraemia [16] and rapid CD4 T‐cell loss [17], progresses to clinical latency with low‐level viral replication and gradual immune decline, and ultimately leads to AIDS characterized by severe immunodeficiency. Acute HIV infections often have non‐specific symptoms, including fever, rash and headache [15, 16, 17, 18]. Furthermore, the earliest biomarker detectable by the fourth‐generation enzyme immunoassays appears around 2–3 weeks post‐contraction, complicating the diagnostic process [6]. This delay often leads to acute HIV infection being mistaken for other conditions. There are many cases in which symptoms of acute HIV infections are confused with intervention side effects or other infections [15].

Unlike the current gold standard diagnostic assay, which is the fourth‐generation enzyme immunoassay, NAAT can facilitate the diagnosis of acute HIV infections, as HIV nucleic acid biomarkers are detectable only one week after contraction [15]. In areas with low HIV prevalence, the inclusion of HIV NAAT into screening algorithms can minimize false positives and possible HIV misdiagnoses based on serological tests alone. These tests are especially important in resolving indeterminate serological results, such as those with low signal‐to‐cutoff ratios, and help differentiate between early HIV infection status and nonspecific background reactivity. The very high sensitivity of NAAT tests makes false negative results very rare; however, they can still occur due to the high frequency of HIV mutations [18]. Dual‐target tests, resolve this issue as they can detect HIV‐1 even if a mutation exists in one of the two target sections of the HIV‐1 genome, such as pol, gag, or LTR, and can help to reduce this low risk of false‐negative HIV‐1 NAAT screening results even further [18]. Improper laboratory practices can easily lead to false positives with NAAT technology, however, primarily due to contamination from polymerase chain reaction (PCR) product carryover and contamination in samples or reagents.

Several studies have highlighted the potential of NAAT to improve the identification of acute and primary HIV infections in a variety of populations [19, 20, 21, 22, 23, 24, 25] (Table 1). The Italian study (Raccagni et al. 2022) showed that NAAT detected four acute and one primary HIV infection among 184 high‐risk men who have sex with men (HR‐MSM), indicating an increased detection rate of HIV cases [19]. Similarly, despite a small number of false positives, an observational study in North Carolina identified 23 acutely infected individuals with NAAT out of 109 250 at‐risk patients [20]. Adding HIV‐RNA testing to first‐generation methods increased case detection by as much as 4.1% in a retrospective study conducted in Florida, Los Angeles and New York [21]. In the Dallas study, 148,888 specimens from patients who had come to the sexually transmitted infection clinics and requested an HIV test were screened for HIV [22]. NAAT was integrated into the daily HIV testing algorithm, and 173 Acute HIV Infections were picked up using the upgraded algorithm [22]. In a substudy, out of 64 acute HIV specimens, 15 (23%) were missed by both third‐ and fourth‐generation EIAs and could only be identified using NAAT [22]. The Kenyan study demonstrated the effectiveness of NAAT by identifying one acute infection and one chronic HIV infection for every 750 patients tested [23]. Similarly, among 9280 HIV‐negative or discordant patients, the Malawian study discovered a 0.9% increase in case identification by utilizing NAAT [24].

**TABLE 1:** Summary of studies on nucleic acid testing (NAAT) for acute HIV infection detection.

Study	Cohort	Findings
Italian study (Angelo Raccagni et al., 2022) [19]	184 high‐risk men who have sex with men (HR‐MSM) in IRCCS San Raffaele Teaching Hospital	NAAT identified four acute and one primary HIV infection (more new HIV infections through NAAT identified per 37 patients tested)
North Carolina observational study (Christopher Pilcher et al., 2005) [20]	109,250 at‐risk patients tested in 2002–03 in the state of North Carolina	23 acutely infected individuals were determined, and there were two false positives
Florida, Los Angeles and New York retrospective study (Pragna Patel et al., 2010) [21]	37 012 patients screened using NAAT after first‐generation testing in New York, Los Angeles and Florida	Adding HIV‐RNA testing increased case detection by 4.1% in Los Angeles, 2.9% in New York and 1.4% in Florida
Dallas study (Brian Emerson et al., 2013) [22]	148,888 tested with HIV using third‐ generation enzyme‐linked immunoassay (EIA) and nucleic acid testing in Dallas (USA) between 2009 and 2012	The inclusion of HIV in the diagnostic algorithm led to the identification of 173 additional acute HIV infections
Kenyan study (Eduard Sanders et al., 2021) [23]	19 464 outpatients aged 18–39 years who were seeking healthcare for symptoms compatible with acute HIV infection	One chronic HIV infection for every 40 patients and one acute HIV infection for every 750 patients tested were identified with the inclusion of nucleic acid testing
Malawian study (Sarah Rutstein et al., 2016) [24]	The study's cohort included 9280 HIV‐negative or discordant patients in Lilongwe.	The use of nucleic acid amplification testing identified 59 individuals with acute HIV infection, resulting in a 0.9% increase in case identification

*Note*: The table shows the study name, profile of the cohort recruited and study findings. All studies illustrate that NAAT can identify more true positive acute and chronic HIV‐1 cases.

These results point to NAAT as a major improvement in HIV early detection, which is essential for prompt intervention and treatment. Interestingly, the findings of the Italian study suggest that focusing NAAT on high‐risk groups like HR‐MSM is a highly cost‐effective strategy [19]. In addition to improving cost‐effectiveness, tar‐ geted testing of high‐risk individuals improves diagnostic accuracy by minimizing false positives because NAAT generally has low specificity but high sensitivity. To estimate the risk of acute HIV infections in high‐risk populations, variables such as medical history, social determinants, and relevant symptoms can be evaluated [19, 24].

Accumulating evidence shows that diagnosis of acute HIV infection is helpful to individual patients and a significant global health benefit, as HIV is highly transmissible during acute infection. Beginning antiretroviral therapy (ART) during an acute infection has several positive clinical consequences, such as enhanced immune function preservation, a notably shorter time to viral suppression and a smaller viral reservoir, all of which may be crucial for curative strategies. Early cART initiation facilitated by acute infection diagnosis reduces the risk of virological failure, attrition and mortality [26]. The CD4 count is greater in patients initiating cART earlier rather than later. One study illustrated that patients starting ART within 4 months of HIV acquisition have a significantly higher likelihood of recovering CD4 counts to ≥900 cells/μL compared with those who initiated ART later [27]. Furthermore, those who start cART late have a larger proviral reservoir associated with a greater potential for reactivation upon treatment interruption or failure [28]. High rates of secondary transmission typically characterize the acute phase [29] due to peaked VL levels [16] and patients being unaware of their HIV status. Therefore, incorporating NAAT into the testing algorithm for diagnosing acute infections would substantially reduce HIV transmission rates.

There is a strong drive towards more widespread POC NAAT, as it can facilitate early diagnosis, including acute infection, faster than centralized testing, leading to reduced transmission and more effective intervention of disease progression. Diagnosing acute infections through NAAT is especially critical in the era of pre‐exposure prophylaxis (PrEP) [30]. PrEP is a preventative measure for HIV‐negative, high‐risk individuals. It can be taken in 3 ways: (1) daily oral tablets such as tenofovir and emtricitabine, (2) event‐driven tablets, and (3) long‐acting injectable agents. If taken appropriately, it has been shown to reduce the risk of HIV transmission by over 90% [31, 32]. Drug‐resistant HIV strains could emerge in people who begin PrEP during acute HIV infection when the virus is rapidly replicating but not yet detectable [33]. This is a result of antiretroviral drugs in PrEP not fully suppressing the VL during the acute phase, leading to the emergence of resistance [30]. The evidence behind this is well established; for example, in a study involving six macaques infected with Simian‐Human Immunodeficiency Virus and treated with long‐acting PrEP (cabotegravir) after confirmed infection but before seroconversion, the virus continued to replicate despite plasma drug levels being maintained above therapeutic thresholds, and cabotegravir resistance mutations emerged [33]. Clinical research has also demonstrated that using PrEP during an undiagnosed acute HIV infection increases the risk of drug resistance, as demonstrated by instances in which people who began PrEP with an undiagnosed acute HIV infection rapidly developed resistance mutations to tenofovir and emtricitabine [30]. Therefore, patients beginning PrEP could greatly benefit from NAAT testing as they are generally at a high risk of contracting HIV, and ruling out acute HIV infection before PrEP initiation stops the emergence of cART‐resistant strains [30]. The widespread dissemination of long‐acting injectable PrEP, including 2‐monthly cabotegravir and, most recently, 6‐monthly lenacapavir, will make early diagnosis even more challenging and exacerbate this issue as plasma RNA VL testing is required as a confirmatory test. POC testing will allow for a same‐day initiation of PrEP according to updated treatment algorithms [34].

Nucleic acid amplification testing is currently used to diagnose infants under 18 months, as maternal antibodies can persist for over a year, and cART treatment reduces the sensitivity of standard RNA assays [8]. It has been reported that less than half of infants exposed to HIV in Western and Central Africa received an HIV test before 8 weeks of age [8]. However, over 160,000 infants acquired HIV, and over 100,000 died from HIV‐related causes, according to a WHO report in 2021 [8]. Timely diagnosis in infants is crucial for initiating ART early, improving long‐term outcomes by preventing clinical progression, reducing viral reservoirs and enhancing CD4 T‐lymphocyte recovery [8]. Early infant diagnosis with POC NAAT, if it can be made widely accessible, can have a significant impact on HIV outcome and potential for cure, as there have been documented cases of remission in infants who start cART early, such as the Mississippi baby [35]. Poor adherence to cART in children and adolescents has been a major obstacle in effective control of infection, and timelier POC NAATs could help to improve adherence follow‐up in these populations.

Extending POC NAAT to adults could also significantly benefit global HIV care and prevention by enabling earlier detection, facilitating timely treatment, reducing transmission rates and improving health outcomes. Integrating NAAT into diagnostic protocols could enhance the effectiveness of HIV prevention and control efforts worldwide.

## HIV TREATMENT MONITORING

HIV drug resistance (HIVDR) is a growing problem, particularly in low‐ and middle‐income countries (LMICs). Before integrase inhibitors (INSTIs) were introduced into the first‐line cART regimen recommended by WHO for LMICs in 2019, up to 69% of HIV‐positive infants were resistant to first‐line cART [8]. Acquired HIV drug resistance can arise from inconsistent medication adherence, sub‐optimal drug levels or inadequate regimen potency, leading to incomplete suppression of VL and exposure to selective pressure [7]. Therefore, improved treatment monitoring is critical to avoid the emergence and spread of HIV strains resistant to the newly recommended first‐line cART regimen for LMICs.

Peripheral plasma HIV‐1 RNA, also called VL, is the most widely used biomarker for monitoring the response to cART. The pre‐ART VL and the rate at which the VL declines after cART initiation estimate the likelihood of disease progression [33]. To establish a baseline, the British HIV Association (BHIVA) advises doing peripheral plasma VL testing at the time of initial diagnosis [36]. It also suggests repeating VL testing during follow‐up visits in months 1, 3 and 6 [36]. If VL is not suppressed by at least 10‐fold in the first month, testing is repeated in the second month [36]. BHIVA advises routine VL testing every 6 months if the patient's VL is not controlled after 6 months. For patients who have been started on ART which includes a protease inhibitor (PI), routine testing should be done every 12 months [36]. Testing a different sample is advised if a VL rebound exceeds 500 copies/mL; testing the same sample again is not appropriate [36]. When viraemia is proven, the underlying cause of viraemia should be investigated to prevent the accumulation of drug resistance [37]. In addition to ART resistance, non‐adherence to the treatment regimen should be investigated as a potential factor [36].

HIV drug resistance is strongly influenced by the drug regimen a patient is on [37]. HIVDR testing can be done genotypically by sequencing the therapeutic molecular targets or phenotypically by evaluating the sensitivity of cells to drugs in a culture setting [37]. Sequencing of peripheral blood mononuclear cells (PBMC)‐associated HIV‐1 DNA may identify more potential HIVDR mutations than plasma HIV‐1 RNA sequencing and might offer additional guidance to clinicians for the most appropriate drug regimen to switch a patient onto. Patients with low‐level viraemia or those who are virologically suppressed may benefit from proviral DNA genotypic resistance testing. However, as there is less confirmation on the utility of this approach than there is for resistance testing of plasma viruses, results from proviral DNA genotype resistance testing should thus be interpreted cautiously [36].

Treatment modifications are required to sustain viral suppression after resistance to the first cART regimen is identified. Usually, one INSTI, such as dolutegravir or bictegravir, and two nucleoside reverse transcriptase inhibitors (NRTIs), such as tenofovir disoproxil fumarate or tenofovir alafenamide, are combined with emtricitabine or lamivudine in the first‐line cART regimen [37]. If resistance is found, the treatment regimen is altered to a second‐line combination, which entails choosing two NRTIs distinct from the first‐line regimen and a pharmacologically boosted PI, such as darunavir/ritonavir or atazanavir/ritonavir. If patients are not responding to the second‐line cART regimen, there are several options from six different antiretroviral classes [37]. The development of more suitable POC VL testing platforms, particularly for low‐resource settings, will be invaluable for picking up rising VL in a timelier fashion so that adherence interventions can be initiated more efficaciously. Currently, in LMICs, it can take several months before patients with high VL are retested and counselled regarding adherence or moved over to second‐line therapy.

**FIGURE 1:**
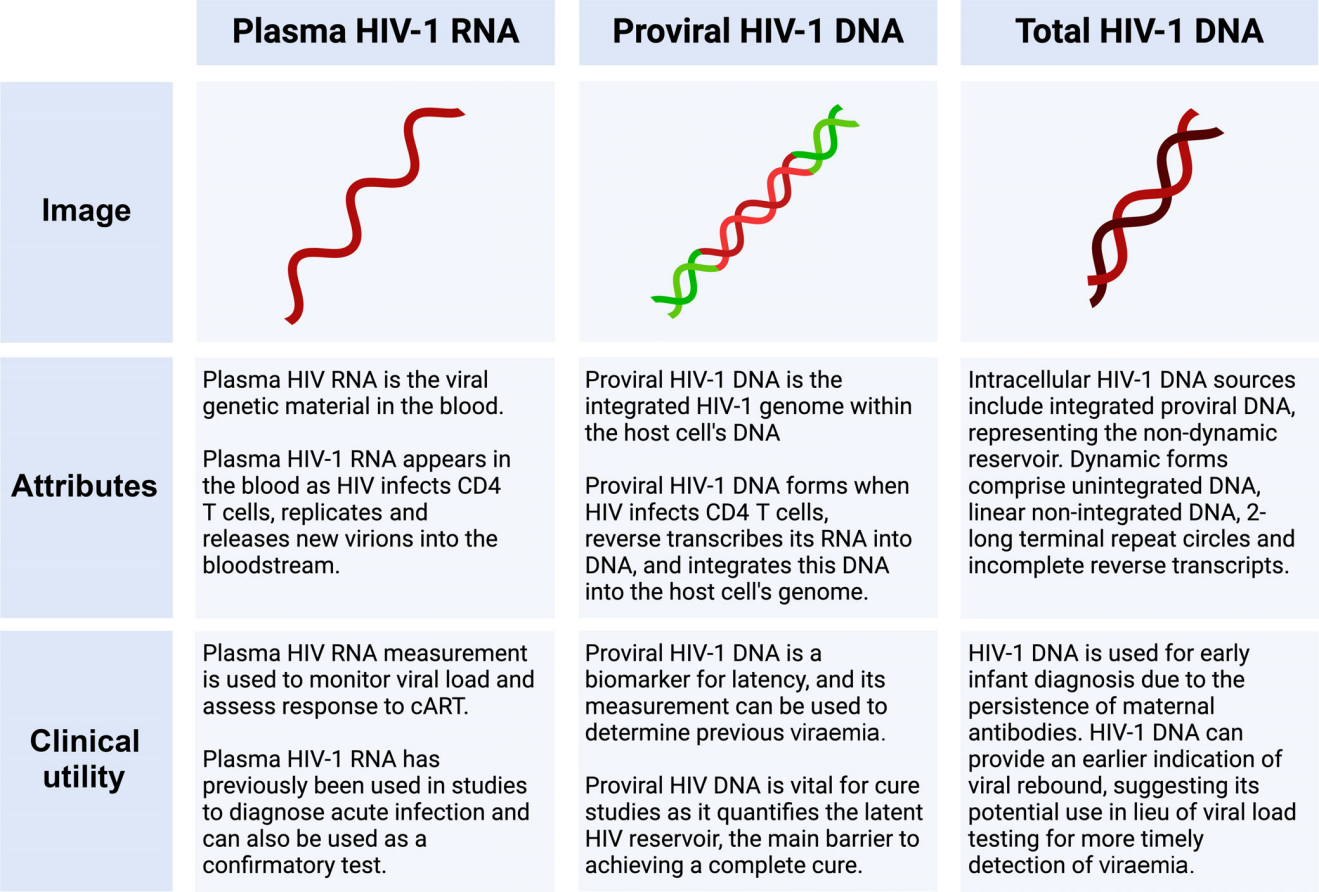
Biomarkers for understanding HIV‐1 infections, including plasma HIV‐1 RNA, proviral HIV‐1 DNA, and total HIV‐1 DNA. (created by https://www.Biorender.com)

Monitoring of HIV‐1 DNA within PBMCs in individuals on cART who are virologically suppressed may be useful as an early indicator of the likelihood of escape from suppression and to identify viral rebound earlier than plasma HIV RNA [38]. The SPARTAC trial has shown that total HIV‐1 DNA is a stronger predictor of viraemia than plasma VL [38]. While integrated proviral DNA is associated with latency, other forms of cell‐associated HIV‐1 DNA, such as unintegrated DNA, linear non‐integrated DNA, 1‐ or 2‐long terminal repeat (LTR) circles and incomplete reverse transcripts, are linked to recent infections and active viral replication (Figure 1). Furthermore, measuring total HIV‐1 DNA in PBMCs allows for more convenient sample preparation and is more affordable than plasma VL, making it better suited for use in POC settings [10, 39]. Current POC NAAT testing platforms detect total HIV DNA but cannot quantify VL or be used for treatment monitoring, as this is based on plasma HIV RNA.

## NAAT TESTING PLATFORMS AND TURNAROUND TIMES

Nucleic acid amplification testing consists of highly sensitive PCR‐based assays that detect HIV RNA or DNA in blood and are typically performed on fully automated platforms for sample processing, amplification and detection. Monitoring of VL (quantitative NAAT) following therapy is typically performed in centralized public laboratories on automated platforms such as the Real‐time HIV‐1 (Abbott Molecular, Des Plaines, Illinois, USA); the COBAS® AmpliPrep/COBAS® TaqMan® HIV‐1 Test v2.0 (Roche Molecular Diagnostics, Basel, Switzerland); and the Hologic Aptima® HIV‐1 Quant Dx Assay on the Panther platform [10, 40, 41]. However, these tests are expensive, require cold chain, require specialist equipment and cannot be provided at the POC [10, 40, 41]. Centralized testing using these quantitative NAAT assays resulted in average turnaround times of 51 days in rural Uganda [42] and 67 days in rural Zimbabwe [40].

**FIGURE 2:**
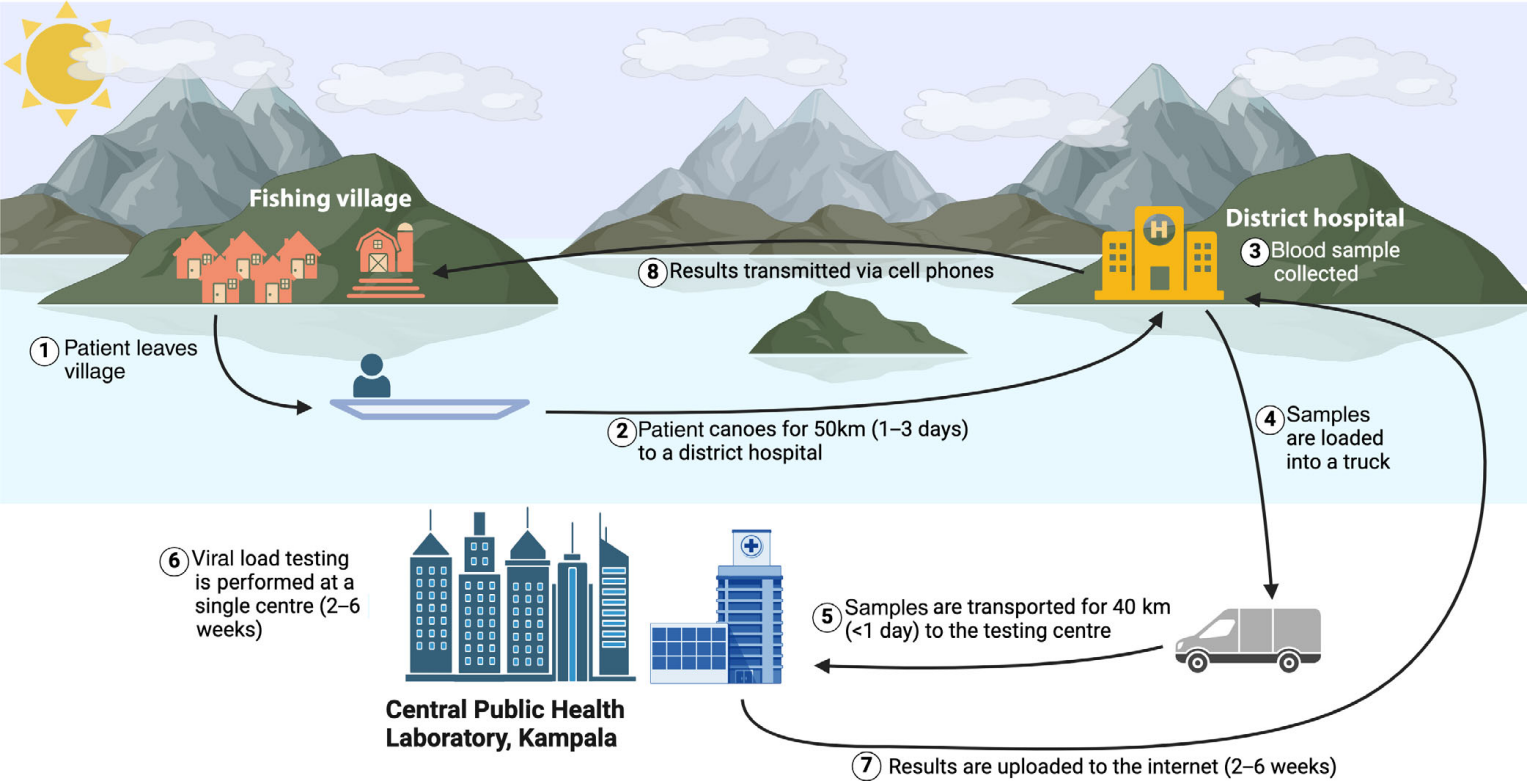
Steps required for HIV patients to undertake viral load testing and receive results in an HIV‐1‐prevalent remote Ugandan fishing village (created by Biorender.com). (1–2) Patients must leave their home village and canoe a long distance to reach a district hospital. (3) Blood samples are collected from patients in the district hospital. (4–5) Samples are loaded onto a lorry and transported inland to the capital city, Kampala. (6) Viral load testing is performed at a central laboratory in Kampala. (7) Results are sent back to the district hospital by uploading the results to the internet. (8) The district hospital relays the results back to the patients via mobile phone.

Access to centralized testing may present significant obstacles to individuals seeking HIV testing, monitoring or treatment in many parts of sub‐Saharan Africa and other LMICs where HIV is prevalent. In a typical remote fishing village in Uganda, for example, a patient may need to canoe for 50 kilometres, taking 1–3 days, to reach a district hospital where their blood sample is collected (Figure 2). The sample is then transported by lorry to the Central Public Health Laboratory in Kampala for testing, and the results are transmitted back via mobile phones to the district hospital and then to the patient [10, 11, 12, 40, 41, 42]. This fishing village, like most others in Uganda, currently does not have mobile phone reception. Delays in obtaining results thus lead to the average turnaround time of 51 days in rural Ugandan settings [42].

For this reason, research has concentrated on creating POC quantitative NAAT [10]. Using individual cartridges for integrated extraction and quantitative real‐time PCR, the Cepheid Xpert HIV‐1 Viral Load system is a POC assay that streamlines VL testing. This enables minimally trained staff to provide same‐day results at health facilities. This POC HIV‐1 RNA testing has been established as the standard of care in assessing HIV‐positive patient prognosis, and clinical decisions can be made more quickly because fewer logistical issues related to traditional VL testing are present, such as the need for central laboratories and specimen transportation [10, 11, 12, 40, 41, 42]. Currently, the Xpert HIV VL assay is being used in the US, Europe, sub‐Saharan Africa and other countries recognising the CE mark. Studies in rural communities (after home‐based collection of venous blood) have been very promising, but the need for centrifugation continues to present a challenge to its routine use as a true POC test [40].

With the presentation of the POC Cepheid Xpert HIV‐1 Viral Load system, turnaround times for VL test results were significantly reduced across healthcare settings in sub‐Saharan Africa. In Ugandan rural communities, the introduction of POC VL monitoring services has significantly reduced turnaround times from 51 days to less than a day, enabling timely clinical decision‐making for adjusting antiretroviral regimens when necessary [11]. Additionally, another study in Uganda found the RAPID‐VL intervention, which included clinician training, rapid near‐POC VL testing and same/next‐day telephone delivery of results, further reduced turnaround times from a pre‐intervention average of 73.4 days to just 1 day post‐intervention [42]. Reduced turnaround times in this study significantly increased 1‐year viral suppression rates among participants, from 76.0% to 83.1%, demonstrating its potential to strengthen VL operations within national ART programmes [42].

Additionally, the decentralized HIV care facilitated more efficient management of treatment failure in Malawi [13]. Among suspected treatment failure cases, follow‐up VL testing rates were higher, and the rate of switching to second‐line ART was significantly higher with POC NAAT (86%) compared with NAAT at a single centre (67%). The time to switch to a second‐line regimen was also shorter with POC NAAT (6.8 vs. 9.7 months). Post‐switch to the second‐line regimen, 79% of patients achieved VL suppression [13].

These findings highlight the potential of POC NAAT to improve clinical outcomes by providing timely results and enabling faster clinical decisions. By eliminating logistical obstacles and offering prompt, accurate results, POC nucleic acid testing such as the Cepheid Xpert HIV‐1 Viral Load system can help close major gaps in HIV management and improve patient outcomes in Sub‐Saharan Africa [40].

## DISCUSSION

HIV has posed a significant burden on global health since the beginning of the epidemic, with 85.6 million infections worldwide. To effectively address the HIV epidemic, it is projected that US$20.8 billion will be required annually by 2025 for the global HIV response [3]. Despite extensive effort, the development of a safe and effective HIV‐1 vaccine continues to be thwarted by challenges of the extensive genetic variability of the virus, the rapid establishment of persistent viral latency, and the limitations in induction of broadly neutralizing antibodies [43]. The focus, therefore, must remain on the management and diagnosis of HIV. VL suppression to undetectable levels through cART is long established to prevent the transmission of HIV; therefore, the HIV epidemic can be significantly suppressed through improved access to cART, VL monitoring and diagnostics [43].

The Joint United Nations Programme on HIV/AIDS (UNAIDS) has set the 95‐95‐95 global target for 2025 in which 95% of all people living with HIV should know their status, 95% of these should be receiving sustained ART, and 95% of those under therapy should have suppressed VLs [10, 44]. Reaching the 95‐95‐95 target will help to control the HIV epidemic [10, 44]. Most Southern African countries, where HIV is highly prevalent, however, are far from these targets. It is estimated that in 2022, only 87% of people with HIV were aware of their status in these countries, only 83% of whom were on antiretrovirals, and just 44% of those on antiretrovirals in these countries had access to VL testing, meaning it is highly likely that less than 95% of patients on cART had suppressed VL levels [10].

Rwanda, located in East‐Central Africa, is a rare example of an HIV‐prevalent low‐income country to achieve the 95‐95‐95 targets [44]. Healthcare decentralization in Rwanda has significantly contributed to achieving the 95‐95‐95 targets by improving access to VL monitoring and diagnostics, enabling timely treatment and better management of HIV at the community level [44]. Achieving the 95‐95‐95 targets in Rwanda has led to significant reductions in HIV transmission, improved health outcomes and cost savings [44].

To achieve and exceed 95‐95‐95 targets, better screening programmes must be introduced, including access to NAAT for acute infection diagnosis in high‐risk populations, so that more patients are aware of their status. HIV transmission is greater during the acute phase because patients are usually unaware of their status, and the VL peaks in the blood and secretions [16, 19, 20, 21, 22, 23, 24, 25]. Early POC NAAT of HIV offers enormous benefits. Early HIV testing allows for rapid treatment, resulting in significant public health benefit by minimizing the risk of spread and transmission (*R*_0_) [45]. Although there is a strong rationale for initiating ART during acute and early HIV infection, early diagnosis and treatment are difficult, and the window of opportunity is limited; however, the rapid evolution of HIV‐1 POC NAAT, including the detectability of HIV RNA (Fiebig I), can avert individuals, reduce *R*_0_ and prevent transmission. Broadly applicable criteria are required to conduct research that represents the variability of both the virus and affected populations, including the geographic diversity of HIV subtypes, which may alter *R*_0_, disease pathogenesis and outcomes. Patients being aware of their status during the acute phase is especially critical. By introducing NAAT, early detection and subsequent treatment can significantly reduce the VL in individuals, thereby lowering the transmission rate [29, 45]. This reduction in transmission can decrease the basic reproduction number (*R*_0_), potentially bringing it to less than one, a threshold associated with controlling and ultimately ending epidemics [45].

Equitable access to VL monitoring services, facilitated by the implementation of POC diagnostic technologies, particularly in under‐served remote settings, will ensure faster test turnaround times [10, 11, 12, 40, 42]. This will allow for timely adjustments to combination cART, driving virological suppression in a greater proportion of HIV patients [13, 36, 37]. This will eventually lead to untransmissible infection and improved health outcomes [8].

Furthermore, EIA can provide false positives or invalid results for particular populations, making NAAT a crucial confirmatory test. As an example, the University of Queensland's COVID‐19 vaccine candidate, which employed molecular clamp technology with elements from HIV gp41 to stabilize the spike protein of SARS‐CoV‐2 in the vaccine construct, was halted after Phase I trials due to unexpected false‐positive HIV test results stemming from the vaccine‐triggered immune response against gp41 [46, 47]. Prophylactic HIV vaccines are designed to stimulate the immune system to produce antibodies against HIV. However, these vaccine‐induced antibodies can cause false‐positive results on EIA tests [47, 48]. Furthermore, PrEP can lead to invalid HIV test results due to suppressed VL and delayed antibody response, which complicates the detection of acute HIV infection and may result in missed diagnoses and drug resistance [29].

In addition to POC VL assays, such as the Cepheid Xpert HIV‐1 Viral Load system, novel VL assays that do not require instruments like thermocyclers have been developed [49]. Reverse transcription loop‐mediated isothermal amplification (RT‐LAMP) is a rapid, highly specific and sensitive technique for detecting RNA by amplifying it under isothermal conditions [49]. The RT‐LAMP assay is practical and simple to perform; it requires minimal equipment and can be completed with basic training.

In conclusion, approximately 65% of people living with HIV in 2023 lived in sub‐Saharan Africa. This region currently accounts for 84% of children and adolescents living with the disease worldwide. The majority of them live in rural or resource‐constrained settings where access to early diagnosis and efficient treatment monitoring is limited. Finding avenues to integrate POC NAAT into these communities, in particular, will bring healthcare systems in these highly burdened regions closer to achieving the UNAIDS 95‐95‐95 goals by ensuring rapid diagnosis and effective monitoring of VL. Decentralization of HIV care through POC NAAT reduces turnaround times, allows for timely adjustments in cART and facilitates the achievement of undetectable VL levels, which correspond to untransmissible infection. Furthermore, incorporating NAAT for acute infection diagnosis is crucial, as it enables early detection and treatment during the highly infectious acute phase, significantly reducing transmission rates. POC NAAT implementation has the potential to decrease the *R*_0_ of HIV transmission, a critical threshold for controlling and suppressing the HIV epidemic. Expanding equitable access to these diagnostic technologies in all communities is essential for meeting global HIV targets, improving health outcomes and reducing transmission rates.

## AUTHOR CONTRIBUTIONS

SSP initiated and drafted the manuscript with supervision, edits and reference suggestions from CK and NI.MM critiqued, restructured and edited the manuscript, providing further reference suggestions and up to 15% of additional text. SS critiqued, edited and provided a few critical references. CK and SSP edited and coordinated the completion of the manuscript.
